# Influence of chronic kidney disease on anticoagulation levels and bleeding after primary percutaneous coronary intervention in patients treated with unfractionated heparin

**DOI:** 10.1007/s11239-015-1255-x

**Published:** 2015-08-04

**Authors:** Wouter J. Kikkert, Peter M. van Brussel, Peter Damman, Bimmer E. Claessen, Jan P. van Straalen, Marije M. Vis, Jan Baan, Karel T. Koch, Ron J. Peters, Robbert J. de Winter, Jan J. Piek, Jan G. P. Tijssen, Jose P. S. Henriques

**Affiliations:** Department of Cardiology, Academic Medical Center, University of Amsterdam, Meibergdreef 9, 1105 AZ Amsterdam, The Netherlands; Department of Clinical Chemistry, Academic Medical Center, University of Amsterdam, Amsterdam, The Netherlands

**Keywords:** Unfractionated heparin, Acute myocardial infarction, Chronic kidney disease, Hemorrhage, Percutaneous coronary intervention

## Abstract

Unfractionated heparin (UFH) plasma protein binding and elimination might be impaired in patients with chronic kidney disease (CKD—defined as creatinine clearance <60 ml/min). It is currently unknown at which UFH bolus dose persistent prolongation of activated partial thromboplastin time (aPTT) occurs in ST-segment elevation myocardial infarction (STEMI) patients with CKD. We investigated the effect of different UFH bolus doses on the first aPTT measured within 6 and 12 h after PPCI in 1071 STEMI patients with and without CKD undergoing primary percutaneous coronary intervention (PPCI) between 1-1-2003 and 31-07-2008. In the first 6 h after PPCI, aPTT ratio was 5.1 for patients with CKD versus 3.4 for those without (p < 0.001). The proportion of patients with markedly high aPTTs (aPTT ratio ≥ 4 times control) increased with increasing heparin bolus and beyond 130 IU/kg there was a marked difference between patients with and without CKD (74.1 and 42.3 % respectively, p < 0.001). By multivariable analysis, CKD was associated with an increased risk of markedly high aPTTs (odds ratio (OR) 2.04; 95 % confidence interval (CI) 1.27–3.27), driven largely by an increased risk of aPTT prolongation in patients treated with UFH boluses ≥130 IU/kg (OR 3.69; 95 % CI 1.85–7.36; p for interaction = 0.009). In conclusion, CKD is associated with severe persistent aPTT prolongation in STEMI patients undergoing PPCI, possibly due to impaired plasma protein binding and reduced UFH elimination. A lower heparin bolus dose might result in lower aPTTs and less bleeding complications in patients with CKD undergoing PPCI.

**Electronic supplementary material:**

The online version of this article (doi:10.1007/s11239-015-1255-x) contains supplementary material, which is available to authorized users.

## Introduction

Chronic kidney disease (CKD) is associated with increased mortality and major bleeding in patients undergoing primary percutaneous coronary intervention (PPCI) for ST-segment elevation myocardial infarction (STEMI) [1, 2]. Therefore, considerable effort has been made to investigate the optimal adjunctive antithrombotic therapy to suppress both bleeding complications as well as recurrent ischemic outcomes in these patients. The European Society of Cardiology (ESC), American Heart Association (AHA) and American College of Cardiology (ACC) currently recommend the use of unfractionated heparin (UFH),enoxaparin or bivalirudin in STEMI patients undergoing PPCI [3, 4]. Of these 3 agents, UFH is currently the most commonly used anticoagulant for this indication [5, 6]. UFH was recently shown to be associated with lower rates of thrombotic events and similar bleeding events as compared to bivalirudin in a large scale all-comer randomized controlled trial reflecting contemporary practice (i.e. bail-out GP IIb/IIIa inhibitor use, radial access PCI and use of novel P2Y12 inhibitors) [7]. Therefore, UFH has gained renewed attention, and considering the low costs associated with its use, UFH will likely remain the most broadly applied anticoagulant in the routine clinical setting of PPCI for years to come. However, despite the extensive experience with UFH, the optimal UFH bolus dose during PPCI remains unknown, especially in patients with CKD.

The intensity of a drug’s action is dependent on the free unbound concentration in the blood. Since plasma protein binding of anionic drugs is impaired in patients with renal failure, one can hypothesize that the unbound fraction of heparin (an anionic drug) after a bolus is increased in patients with renal failure, resulting in prolonged aPTTs. In addition, pharmacokinetic studies have shown that in low doses UFH is cleared by binding to endothelial cell receptors and by depolymerization by macrophages [8]. In high doses, clearance by endothelial cells and macrophages is saturated and additional clearance occurs by the kidneys [9, 10]. Therefore, we hypothesized that the elimination of UFH in patients with CKD might be reduced and that administering repeated boluses to patients with CKD might result in prolonged UFH half-life and excess anticoagulation. Therefore the aim of the current analysis was to compare aPTT values within the first 12 h after PPCI in response to different UFH bolus doses for patients with and without CKD.

## Methods

### Source population and procedures

The data analyzed in this study were obtained from STEMI patients accepted for PPCI at the Academic Medical Center–University of Amsterdam between January 1, 2003, and July 31, 2008. The study complied with the Declaration of Helsinki, and the local ethics committee approved the study protocol. In general, patients qualified for PPCI if they had typical ischemic chest pain and at least 1 mm ST-segment elevation in 2 or more contiguous leads, a new left bundle-branch block, or a true posterior myocardial infarction. Patients received a standard 300–600 mg loading dose clopidogrel. If a coronary stent was implanted, clopidogrel was prescribed for at least 1 month to patients with a bare metal stent and for 6–12 months to patients with a drug-eluting stent. Patients were routinely pretreated with 300 mg aspirin and 5000 IU unfractionated heparin, either in the ambulance during transfer to the catheterization laboratory, or in the referral hospital. An additional heparin bolus was administered at the catheterization laboratory if necessary, to maintain an aPTT approximately two times the upper limit of normal (ULN). This was followed by an infusion of 12 IU/kg/h, which was maintained up to 24–48 h after the procedure by our center’s clinical protocol. APTT monitoring was required to be performed approximately every 6 h. Adjustments to the infusion rate were subsequently made according to a dose nomogram which was derived from the standard ACC/AHA weight based dose-nomogram [11]. Glycoprotein IIb/IIIa inhibitors (GPIs) were used in a bail-out setting at the discretion of the operator. Patients treated with a GPI generally received a 0.25 mg/kg bolus abciximab intravenously followed by an 0.125 µg/kg/min intravenous infusion for up to 12 h after the procedure, after which heparin therapy was reinitiated.

All laboratory assessments (including aPTT) were stored in the institutional laboratory database. Procedural and angiographic data were prospectively collected and entered by interventional cardiologists and specialized nurses in a dedicated database. Chart review for consecutive STEMI patients with available aPTT measurements was performed in the context of a study designed to investigate the relationship between aPTT and clinical outcome in STEMI patients treated with PPCI. A detailed description of the study protocol and study population has been previously published [12, 13].

Laboratory measurements (including aPTT, serum creatinine and hemoglobin) which were measured in referring hospitals were added to the study database. We obtained clinical history and detailed information on peri-procedural treatment from in-patients records in the PCI center and referring hospitals. We obtained follow-up of clinical outcome, including recurrent MI, stroke, stent thrombosis, revasularization procedures and bleeding, by reviewing in- and outpatients charts in the tertiary PCI center and referring hospitals between 2011 and 2012. For every patient, we systematically checked in-patients charts of every hospital admission for the occurrence of clinical events, including hemorrhagic events and their location. Follow-up of clinical events was censored at the actual date of chart review. Patients whose whereabouts could not be traced were considered lost to follow-up from the date of last known medical contact. Follow-up information regarding vital status was obtained from computerized, long-term mortality records from the National Death Index. If a patient could not be identified in these records (e.g. foreign patients), censoring was at the date of last contact. For the present analysis, patients were censored at the date of death or at hospital discharge, whichever came first.

### Study design

Patients were eligible for inclusion in the analysis if: (1) they had typical ischemic chest pain and at least 1 mm ST-segment elevation in 2 or more contiguous leads, a new left bundle-branch block, or a true posterior myocardial infarction; (2) an aPTT was recorded between arterial sheath insertion and 12 h thereafter and (3) a serum creatinine was recorded pre-procedurally or within 1 h of arterial puncture. We excluded patients who were pretreated with both UFH and low molecular weight heparin (LMWH).

### Measurements and definitions

For each patient we used the first aPTT measured after arterial sheath insertion and categorized these aPTTs in the following time-intervals: from 0 h until 6 h (referred to as: ‘6 h’), from 6 h until 12 h (referred to as: ‘12 h’), and from 0 h until 12 h (referred to as: ‘first 12 h’). All times were expressed relative to the moment of arterial sheath insertion at the start of PPCI. APTT ratios were stratified in the following groups: subtherapeutic (below 1.5 times control), therapeutic: between 1.5 and 2.0 times control; high: between 2.0 and 3.99 times control; and markedly high: ≥4 times control. APTTs are presented in relation to the hospital and reagent specific upper limit of normal. If the electronic laboratory database indicated a value ‘0’ for aPTT, this measurement was treated as not having been performed. In case a patient had no measurements within a given time interval, the value for that time interval was declared missing. The Cockcroft Gault formula was used to calculate creatinine clearance [14]. Baseline creatinine clearance was calculated from the serum creatinine measurement that was closest to the moment of arterial sheath insertion. CKD was defined as creatinine clearance <60 ml/min.

### Outcome definitions

Primary outcome was aPTT within the first 6 h after PCI. Secondary outcomes included aPTT at different time intervals after PCI (specified above), the in hospital occurrence of Bleeding Academic Research Consortium defined bleeding complications, and the occurrence of major adverse cardiac events (MACE) during the initial hospitalization. MACE was defined as the non-hierarchical composite of cardiac mortality, recurrent MI, stroke or target lesion revascularization (TLR). Recurrent MI and TLR were defined according to the Academic Research Consortium criteria [15]. Stroke was defined as an irreversible neurological deficit, as classified by a neurologist, on the basis of supporting information, including brain images and neurologic evaluation.

### Statistical analysis

Normally distributed continuous variables are reported as the mean with standard deviation (SD) and compared with the Student *t* test, skewed distributed variables are presented as the median with interquartile range (IQR) and compared with the Mann–Whitney *U* test. Categorical variables are presented as proportions and compared with the *χ*^2^ test or Fisher’s exact test.

To investigate the relationship between estimated creatinine clearance and aPTT ratio beyond four times control, we performed 3 sets of logistic regression analyses for each peri-procedural timeframe (6, 12 h, first 12 h): (1) unadjusted; (2) adjusted for age, gender, bodymass, length/size, time to first aPTT measurement and heparin bolus dose, and (3) adjusted for relevant predictors of aPTT ratios beyond four times control. Relevant predictors for aPTT ratios beyond four times control were determined per timeframe, using stepwise backward elimination logistic regression analyses, including the following candidate covariables: gender, body mass, length, heparin bolus dose, time to first aPTT measurement, history of hypertension, diabetes, dyslipidemia, current smoking, stroke or TIA, peripheral artery disease, malignant disease, bleeding, recent surgery, previous MI, family history of CAD, anemia, leucocyte count, thrombocyte count, use of GP IIb/IIIa inhibitor, cardiogenic shock, and use of IABP. A covariate was included in the model if it influenced the model with a p < 0.10 by the likelihood ratio test and was removed if its significance level exceeded p = 0.10. To investigate if there was an interaction between UFH bolus dose and creatinine clearance <60 ml/min/m^2^ we included interaction terms between UFH bolus dose and creatinine clearance. The relationship between creatinine clearance and the in hospital occurrence of BARC type ≥3 bleeding, and MACE was investigated by developing two sets of logistic regression models for each outcome: unadjusted and adjusted for relevant predictors of each outcome. Relevant predictors were determined using stepwise, backward elimination logistic regression analyses including all covariables with a significant unadjusted relationship with each outcome (p < 0.10). As a sensitivity analysis we performed an additional analysis including duration of heparin treatment and vitamin K antagonist treatment in addition to the previously identified predictors of bleeding in the multivariable model for BARC type ≥3 bleeding. All tests were 2-sided and a p value below 0.05 was considered statistically significant. All statistical analyses were performed using Statistical Package for Social Sciences software (SPSS version 20.0, Chicago, Illinois).

## Results

Between 1-1-2003 and 31-07-2008, a total of 3472 patients with acute myocardial infarction were admitted to our catheterization laboratory with an indication for PPCI. 1928 patients were excluded because these patients did not have an aPTT measured between the start of PCI and 12 h thereafter or because they were not receiving UFH treatment at the time of the aPTT measurement. Of these 1544 patients baseline creatinine clearance was available in 1332 patients. Of these 1332 patients we had data on heparin bolus in 1185 patients. 114 patients were excluded from the analysis because they were pretreated with LMWH. Therefore, the study cohort consists of 1071 patients, of whom 195 patients (18.2 %) had an estimated creatinine clearance <60 ml/min. 16 patients had an estimated creatinine clearance <30 ml/min, of whom 2 had end stage renal disease (ESRD (defined as CrCl <15 ml/min)). Baseline, procedural and angiographic characteristics for patients in- and excluded in the analysis are given in supplementary Table 1. Patients included in the analysis were less likely to be male, and less likely to be treated with GP IIb/IIIa inhibitors. They more often presented in cardiogenic shock and were more often treated with intra-aortic balloon pump (IABP). One year mortality was 11.7 % in patients included in the analysis, whereas mortality was 10.9 % in patients excluded (p = 0.52).The median total UFH bolus was 125 IU/kg (interquartile range (IQR): 100–154), consisting of a median pre-catheterisation laboratory bolus of 60 IU/kg (IQR 50– 70) and an additional 66 IU/kg (IQR 56–83) given during the catheterisation procedure. In 87.1 % of patients treated with 70–100 IU/kg UFH the aPTT measured within the first 6 h after PPCI was outside the recommended range (between 1.5 and 2.0 time ULN) and in as many as 66.3 % of patients the aPTT was in excess of the recommended range. In 25.7 % of patients aPTT was markedly prolonged (>4 times ULN) after an 70–100 IU/kg UFH bolus dose. Baseline characteristics and treatment strategies by estimated creatinine clearance are presented in Table 1. The aPTT ratio was 3.7 (interquartile range (IQR) 2.1–5.7) in the first 6 h after PPCI, 2.6 (IQR 1.7–4.2) between 6 and 12 h after PPCI and 3.1 (IQR 1.9–5.1) within the first 12 h after PPCI. In the first 6 h after PPCI, aPTT ratio was 5.1 for patients with CKD as compared to 3.4 for those without (p < 0.001). In the 6 h timeframe, aPTT measurements were obtained significantly later in patients with CKD (2.4 versus 2.0 h, p = 0.017). There was no statistically significant difference in time to first aPTT measurement in the 12 h time frame and first 12 h timeframe (8.4 and 6.6 h in patients with CKD as compared to 8.3 and 6.5 h in patients without CKD; p = 0.78 and p = 0.25 respectively).

**Table 1:** Baseline, procedural and angiographic characteristics of the study patients

Characteristic	Creatinine clearance (ml/min)		p value
	<60 (n = 195)	≥60 (n = 876)	
Male, n/N (%)	80/195 (41.0)	655/876 (74.8)	<0.001
Age (years), mean (±SD)	76 (±8.6)	59 (± 11.8)	<0.001
Length (m), median (IQR)	1.67 (1.60–1.75)	1.75 (1.70–1.80)	<0.001
Body mass (kg), median (IQR)	70 (62–80)	81 (74–91)	<0.001
Body mass index, median (IQR)	25.0 (22.6–27.7)	26.3 (24.3–29.2)	<0.001
History of n/N (%)			
Diabetes	35/195 (17.9)	108/876 (12.3)	0.037
Hypertension	84/195 (43.1)	304/876 (34.7)	0.028
Hypercholesterolemia	32/195 (16.4)	206/876 (23.5)	0.031
Current smoking	44/195 (22.6)	443/876 (50.6)	<0.001
Previous stroke or TIA	20/195 10.3)	48/876 (5.5)	0.013
Peripheral vascular disease	30/195 (15.4)	39/876 (4.5)	<0.001
Pre-existent malignant disease	32/195 (16.4)	59/876 (6.7)	<0.001
Recent surgery (<10 days)	5/195 (2.6)	7/876 (0.8)	0.050
Bleeding	19/195 (9.7)	30/876 (3.4)	<0.001
Previous MI	27/195 (13.8)	91/876 (10.4)	0.16
Previous PCI	12/195 (6.2)	69/876 (7.9)	0.41
Previous CABG	2/195 (1.0)	15/876 (1.7)	0.75
Family history CAD	37/195 (19.0)	374/876 (42.7)	<0.001
Laboratory values			
White blood cell count ≥ 11 × 10^9^/l, n/N (%)	94/191 (49.2)	459/865 (53.1)	0.34
Anemia, n/N (%)^a^	73/195 (37.4)	102/875 (11.7)	<0.001
Creatinine clearance, median (IQR)^b^	46.6 (38.7–54.5)	99.4 (79.5–122)	<0.001
Thrombocyte count (×10^9^/l), n/N (%)			0.21
<150	10/193 (5.2)	29/868 (3.3)	
150–400	173/193 (89.6)	810/868 (93.3)	
>400	10/193 (5.2)	29/868 (3.3)	
Total ischemic time (min),median (IQR)	207 (143–297)	182 (130–260)	0.005
Cardiogenic shock, n/N (%)	26/193 (13.5)	60/873 (6.9)	0.002
IABP, n/N (%)^c^	40/195 (20.5)	93/874 (10.6)	<0.001
Loading dose clopidogrel, n/N (%)			0.001
300 mg	116/194 (59.8)	513/865 (59.3)	
600 mg	65/194 (33.5)	333/865 (38.5)	
Other	1/194 (0.5)	6/865 (0.7)	
Glycoprotein IIb/IIIa inhibitor, n/N (%)	23/195 (11.8)	120/876 (13.7)	0.48
Pre-cathlab heparin bolus (IU/kg), median (IQR)	67.6 (55.6–80.6)	58.8 (50.0–67.6)	<0.001
Cathlab bolus dose (IU/kg), median (IQR)	73.5 (61.7–100)	63.3 (54.3–83.3)	<0.001
Total heparin bolus (IU/kg), median (IQR)	134 (106–182)	125 (100–149)	<0.001
Duration of heparin therapy (h), median (IQR)	40.5 (21.0–50.0)	45.0 (20.5–50.5)	0.66
Vitamin K antagonist at discharge, n/N (%)	22/195 (11.3)	72/876 (8.2)	0.17
PCI access site, n/N (%)			0.29
Femoral artery	184/195 (94.4)	835/876 (95.3)	
Radial artery	7/195 (3.6)	34/876 (3.9)	
Other or combinations	4/195 (2.1)	7/876 (0.8)	
Infarct related artery, n/N (%)			0.87
RCA or LCx	109/189 (57.7)	490/859 (57.0)	
LAD or LM	80/189 (42.3)	369/859 (43.0)	
Pre-procedural TIMI flow in IRA, n/N (%)			0.41
0/1	132/177 (74.6)	579/810 (71.5)	
2/3	45/177 (25.4)	231/810 (28.5)	
Post-procedural TIMI flow in IRA, n/N (%)			<0.001
0/1	15/207 (8.1)	14/840 (1.7)	
2/3	170/207 (91.9)	826/840 (98.3)	
Mulitvessel disease, n/N (%)	96/188 (51.1)	273/854 (32.0)	<0.001
Chronic total occlusion, n/N (%)	39/188 (20.7)	93/854 (10.9)	<0.001

*SD* standard deviation, *IQR* interquartile range, *TIA* transient ischemic attack, *MI* myocardial infarction, *PCI* percutaneous coronary intervention, *CABG* coronary artery bypass grafting, *CAD* coronary artery disease, *IABP* intra-aortic balloon pump, *RCA* right coronary artery, *LCx* left circumflex artery, *LAD* left anterior descending artery, *LM* left main artery, *TIMI* thrombolysis in myocardial infarction, *IRA* infarct related artery

^a^Anemia was defined as baseline hemoglobin less than 13 g/dl for males and less than 12 g/dl for females

^b^Creatinine clearance was estimated using the Cockcroft Gault equation

^c^Includes 15 patients who received hemodynamic support with the impella percutaneous left ventricular assist device

### Renal function and anticoagulation with unfractionated heparin

**Fig. 1:**
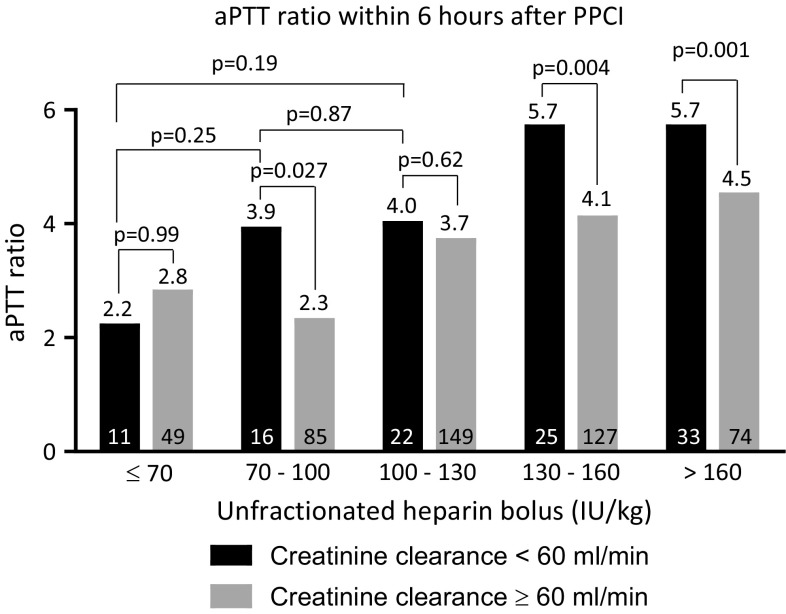
The first aPTT ratio measurement between sheath insertion and 6 h thereafter according to the administered heparin bolus for patients with and without creatinine clearance <60 ml/min. For each heparin bolus dose, the aPTT ratio was outside the recommended range (between 1.5 and 2 times control). Above 70 IU/kg UFH, aPTT was higher in patients with creatinine clearance <60 ml/min. The difference in aPTT ratio seemed to increase with increasing bolus

**Fig. 2:**
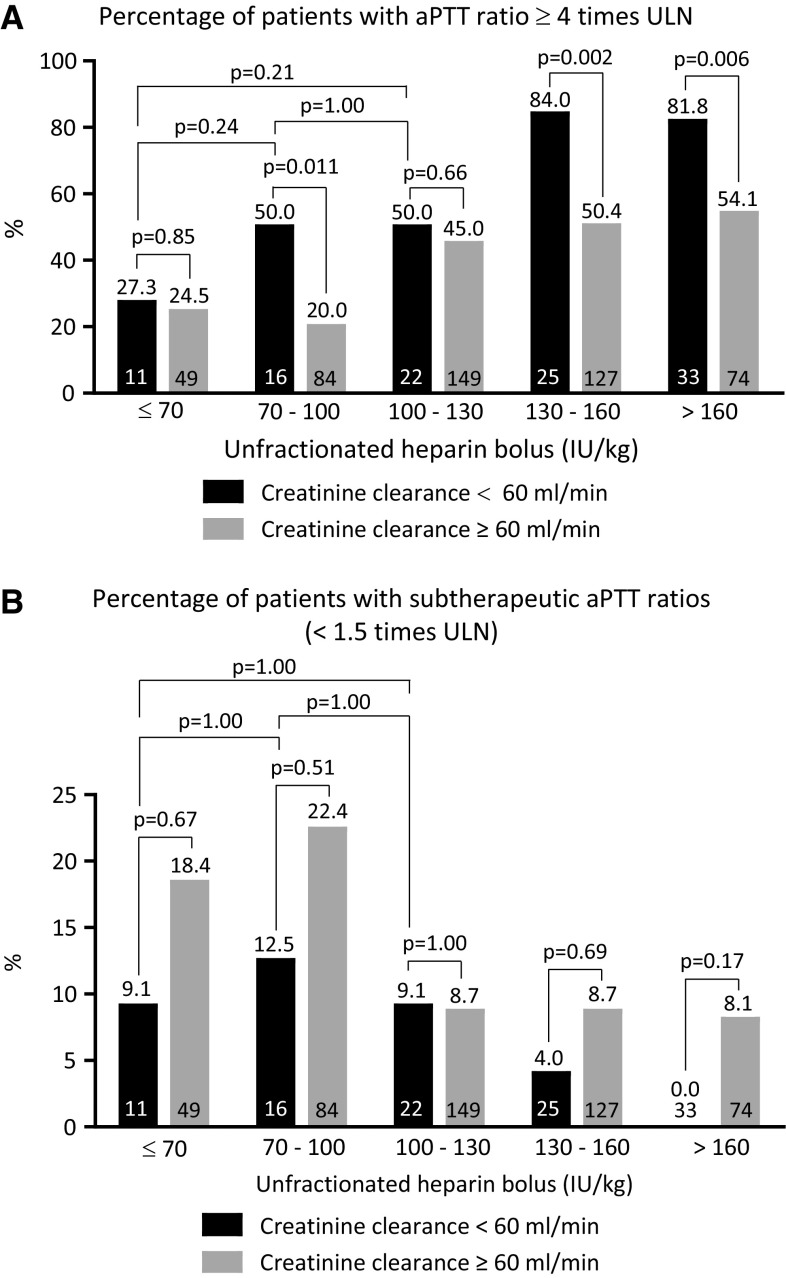
**a** Percentage of patients with a mean aPTT ratio beyond four times control (measured the first 6 h after start of PPCI), as a function of unfractionated heparin bolus dose and creatinine clearance. For each heparin bolus dose, the percentage of patients with a mean aPTT ratio beyond four times control was higher for patients with CKD. The proportion of patients with markedly high aPTTs increased as the heparin bolus increased. The increase in risk of markedly high aPTTs with increasing UFH bolus was greater in patients with CKD, as compare to those without CKD. There was no statistically significant difference in markedly high aPTTs between the patients with CKD treated with a ≤70, 70–100, or 100–130 IU/kg UFH bolus dose (p ≥ 0.54). The *black* and *white numbers* in the *bars* represent the number of patients in the respective patient group. **b** Percentage of patients with a mean aPTT ratio below 1.5 times control (measured within the first 6 h after start of PPCI), as a function of unfractionated heparin bolus dose and creatinine clearance. The proportion of patients with subtherapeutic aPTTs decreased as the heparin bolus decreased. Patients with CKD were less likely to have subtherapeutic aPTTs when boluses in excess of 70 IU/kg were used, although none of the comparisons were statistically significant. ULN indicates upper limit of normal

**Fig. 3:**
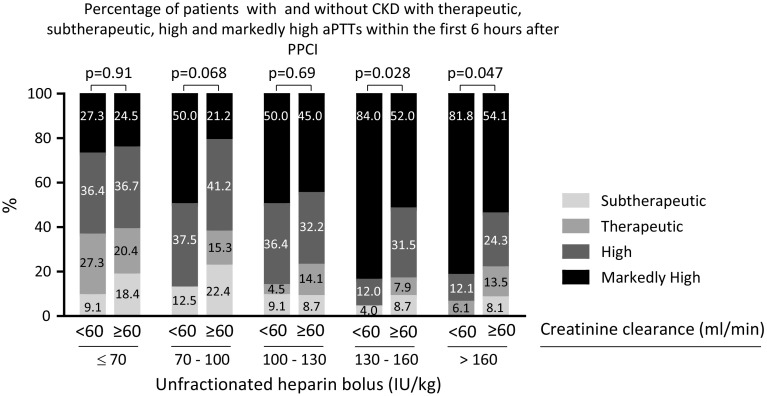
Percentage of patients with therapeutic, subtherapeutic, high and markedly aPTT ratios (measured within 6 h after PPCI), as a function of unfractionated heparin bolus dose and creatinine clearance. Irrespective of the bolus dose heparin, the proportion of patients with an aPTT ratio within the recommended range (1.5–2.0 times control) was low and most of the patients had an aPTT ratio in excess of the recommended range. With increasing heparin bolus dose, the proportion of patients with high and markedly high aPTT ratios increased, whereas the proportion of patients with subtherapeutic and therapeutic aPTT ratios decreased. For patients with CKD, the proportion of patients with therapeutic aPTT ratios was numerically highest when a dose below 70 IU/kg was used, although this proportion was not statistically significant different from the proportion of patients with therapeutic aPTT ratios when a 70–100 or 100–130 IU/kg was used (p ≥ 0.056)

Figure 1 displays the aPTT ratio the first 6  after PPCI for patients with and without CKD according to different categories of UFH bolus. APTT ratios increased with increasing heparin bolus. Above 70 IU/kg, aPTT ratios were higher for patients with CKD and the difference in aPTT ratios between patients with and without CKD seemed to increase with increasing heparin bolus, particularly with heparin boluses in excess of 130 IU/kg. When the currently recommended UFH bolus was used (between 70 and 100 IU/kg), aPTT ratio was significantly higher in patients with CKD. Supplementary Figs. 1 and 2 display aPTT ratios measured between 6 and 12 h after PPCI and the first 12 h after PPCI respectively according to different categories of UFH bolus for patients with and without CKD. Results were similar to aPTT ratios within the first 6 h after PPCI, although the differences between patients with and without CKD were even more prominent. Figure 2a displays the proportion of patients with markedly high aPTTs (≥4 times ULN) for patients with and without CKD according to heparin bolus. The proportion of patients with markedly high aPTT ratios increased with increasing heparin bolus. For every heparin bolus the proportion of patients with excess anticoagulation was higher in patients with CKD. When the recommended bolus was used, the proportion of patients with excess anticoagulation among those with CKD was as high as 50.0 %. Beyond 130 IU/kg the difference between patients with and without CKD who had a markedly high aPTT significantly increased. There was no statistically significant difference in markedly high aPTTs between the patients with CKD treated with ≤70 IU/kg, 70–100 or 100–130 IU/kg bolus dose (p ≥ 0.21). The results were consistent when the relationship between CKD, heparin bolus and aPTT ratio were investigated for the other timeframes (between 6 and 12 h after PPCI and the first 12 h after PPCI; supplementary Figs. 3 and 4 respectively). Figure 2b displays the proportion of patients with subtherapeutic aPTT ratios for patients with and without CKD according to heparin bolus. The proportion of patients with subtherapeutic aPTT ratios decreased as the heparin bolus increased. The proportion of patients with subtherapeutic aPTT ratios tended to be smaller in patients with CKD, as compared to those without CKD. However the differences did not reach statistical significance. Figure 3 shows the distribution of patients with subtherapeutic, therapeutic, high and markedly high aPTT ratios recorded the first 6 h after start of the PPCI for patients with and without CKD according to UFH bolus. Supplementary Figures 5 and 6 show these data for the mean aPTT ratios recorded between 6 and 12 h after PPCI and for the first 12 h after PPCI respectively.

Table 2 provides unadjusted and adjusted odds ratios (ORs) for aPTT beyond 4 times control according to the presence of CKD. CKD was an independent predictor of aPTT beyond four times control. This risk was independent of other components of the Cockcroft-Gault equation of estimated creatinine clearance. The risk of markedly high aPTTs in patients with CKD was mainly driven by the increased risk of markedly high aPTTs when a heparin bolus in excess of 130 IU/kg was administered. To investigate if the increase in proportion of patients with markedly high aPTTs with increasing heparin bolus dose was significantly greater for patients with CKD, as compared to those without, we included an interaction term between heparin bolus and creatinine clearance in the multivariable logistic regression analyses. There was a significant interaction between UFH bolus dose (above or below 130 IU/kg) and CKD, indicating that beyond 130 IU/kg, there was a stronger risk of markedly high aPTTs (measured within the first 6 h and the first 12 h after PPCI) in patients with CKD, as compared to those without.

**Table 2:** Relationship between creatinine clearance <60 ml/min/1.73 m^2^ and aPTT ratio ≥4 times control

Hours after procedure	Creatinine clearance		Unadjusted				Adjusted^a^				p value for interaction^c^	Adjusted^b^				p value for interaction^c^
	<60 ml/min % (n/N)	≥60 ml/min % (n/N)	OR	95% CI		p value	OR	95% CI		p value		OR	95% CI		p value	
6 h^d^	65.4 (70/107)	41.3 (200/484)	2.87	1.73	4.16	<0.001	2.21	1.31	3.72	0.003		2.33	1.43	3.81	0.001	
<130 IU/kg	44.9 (22/49)	33.9 (96/283)	1.59	0.86	2.93	0.14	1.28	0.64	2.56	0.48	0.014	1.12	0.56	2.23	0.76	0.002
≥130 IU/kg	82.8 (48/58)	51.7 (104/201)	4.48	2.15	9.34	<0.001	5.07	2.08	12.4	<0.001		6.31	2.69	14.8	<0.001	
12 h^e^	59.1 (52/88)	21.9 (86/392)	5.14	3.16	8.37	<0.001	1.69	0.92	3.11	0.093		1.48	0.78	2.80	0.23	
<130 IU/kg	45.7 (16/35)	18.6 (42/226)	3.69	1.75	7.77	0.001	1.08	0.43	2.73	0.87	0.25	0.98	0.38	2.54	0.96	0.48
≥130 IU/kg	67.9 (36/53)	26.5 (44/166)	5.87	3.00	11.5	<0.001	2.68	1.15	6.27	0.023		2.14	0.87	5.29	0.10	
First 12 h^f^	62.6 (122/195)	33.0 (289/876)	3.4	2.46	4.69	<0.001	1.98	1.33	2.93	0.001		1.86	1.25	2.78	0.002	
<130 IU/kg	45.2 (38/84)	27.3 (139/509)	2.2	1.37	3.53	0.001	1.19	0.68	2.06	0.55	0.011	1.03	0.58	1.82	0.92	0.003
≥130 IU/kg	75.7 (84/111)	40.9 (150/367)	4.5	2.78	7.28	<0.001	3.66	2.00	6.70	<0.001		3.62	1.95	6.73	<0.001	

*APTT* activated partial thromboplastin time, *CrCl* creatinin clearance

^a^Calculated using multivariable logistic regression analyses adjusting for gender, bodymass, length, time to first aPTT measurement and heparin bolus dose

^b^Calculated using multivariable stepwise backward elimination logistic regression analyses including the following candidate covariables: gender, body mass, length, heparin bolus dose, time to first aPTT measurement, history of hypertension, diabetes, dyslipidemia, current smoking, stroke or TIA, peripheral artery disease, malignant disease, bleeding, recent surgery, previous MI, family history of CAD, anemia, leucocyte count, thrombocyte count, use of GP IIb/IIIa inhibitor, cardiogenic shock, and use of IABP

^c^p value for the interaction term between heparin bolus dose (≥130 versus <130 IU/kg) and creatinine clearance (<60 versus ≥ 60 ml/min)

^d^The first APTT determined between arterial sheath insertion and 6 h hereafter

^e^The firstAPTT determined between 6 and 12 h after arterial sheath insertion

^f^The first APTT determined between arterial sheath insertion and 12 h hereafter

Supplementary Table 2 provides unadjusted and adjusted ORs for aPTT beyond four times control according to estimated creatinine clearance as a continuous variable. Again, creatinine clearance was an independent predictor of markedly high aPTTs, both between 0 and 6 h after PPCI and 6–12 h after PPCI.

### CKD and clinical outcome

Creatinine clearance <60 ml/min was independently associated with in hospital BARC type ≥3 bleeding and MACE (Table 2). Other predictors of in-hospital BARC type ≥ 3 bleeding and MACE are presented in Tables 2 and 3 of the online appendix. In a sensitivity analysis additionally adjusting for duration of heparin therapy and vitamin K antagonist treatment, CKD remained independently associated with in hospital BARC type ≥3 bleeding.

**Table 3:** Relationship between creatinine clearance and in hospital clinical outcome

Outcome	Creatinine clearance	No. (%) of patients	Unadjusted				Adjusted			
			OR	95 % CI		p value	OR	95 % CI		p value
In hospital BARC type ≥3 bleeding										
	<60 (ml/min)	63/195 (32.3)	4.33	2.98	6.29	<0.001	2.78^a^	1.81	4.27	<0.001
	≥60 (ml/min)	87/876 (9.9)	1	–	–	–	1	–	–	–
In hospital MACE										
	<60 (ml/min)	51/195 (26.2)	4.02	2.69	6.00	<0.001	2.52^b^	1.56	4.08	<0.001
	≥60 (ml/min)	71/876 (8.1)	1	–	–	–	1	–	–	–

*OR* odds ratio, *CI* confidence interval, *BARC* bleeding academic research consortium, *MACE* major adverse cardiac event

^a^Calculated using logistic regression analysis adjusting for the use of GP IIb/IIIa inhibitor, intra-aortic balloon counterpulsation, gender, body mass index, and multivessel disease (with or without chronic total occlusion). The results of the multivariable model are given in online supplementary Table 3

^b^Calculated using logistic regression analysis adjusting for family history of coronary artery disease, GP IIb/IIIa inhibitor, intra-aortic balloon counterpulsation, cardiogenic shock, anemia, white blood cell count, thrombocyte count, infarct related artery and multivessel disease (with or without chronic total occlusion). The results of the multivariable model are given in online supplementary Table 4

## Discussion

The main findings of this study can be summarized as follows. After repeated UFH boluses a more persistent aPTT prolongation occurs in patients with CKD as compared to those without CKD. After multivariable adjustments, CKD was an independent predictor of aPTT prolongation. Patients with CKD are particularly at high risk of markedly high aPTTs and bleeding when bolus doses in excess of 130 IU/kg are used.

Several mechanisms may be responsible for the persistent aPTT prolongation in patients with CKD. First, the plasma protein binding of UFH might be reduced in patients with renal failure, thus increasing the free concentration in plasma. The strength of a drug’s action is related to the drug’s peak concentration in plasma. The peak concentration of a drug after an initial bolus is dependent on the bolus dose and the volume of distribution, which in turn is strongly dependent on plasma protein binding. Plasma protein bound drugs are largely inactive. Therefore, reduced plasma protein binding may result in more free drug available at the site of action. The binding of anion drugs to plasma proteins in patients with renal failure is reduced [16]. Thus, the free fraction of unfractionated heparin, an anion drug, might be enhanced in patients with renal failure, explaining the aPTT prolongation in patients with CKD found in the current study. Second, in low and therapeutic doses, heparin is cleared by the reticuloendothelial system [8]. Renal failure is associated with abnormal function of the reticuloendothelial system and impaired function of macrophages [17–19]. Therefore, we hypothesize that clearance of heparin by the reticuloendothelial system might be reduced in patients with CKD. Third, in high doses UFH is cleared by the kidneys [9, 10]. Therefore, accumulation of heparin and its anticoagulant properties may occur in patients with decreased renal function. Fourth, it is possible that a higher prevalence of other, unmeasured causes of aPTT prolongation may have contributed to aPTT prolongation in patients with CKD, such as vitamin K deficiency [20], coagulation factor deficiencies [21], acquired clotting factor inhibitors [22], disseminated intravascular coagulation [23], and massive blood transfusion leading to a dilutional coagulopathy [24]. These disorders however are extremely rare, therefore it is unlikely that imbalances in the prevalence of these disorders among patients with and without CKD are the causative factors for aPTT prolongation.

Consistent with previous studies, CKD was associated with an increased risk of bleeding after PPCI after adjustment for confounders [1]. Possible explanations for the increased bleeding risk among patients with CKD, include functional abnormalities in platelets, abnormal platelet-vessel wall interaction and adverse effects of anemia [25]. The present analysis suggests an additional, modifiable mechanism. We showed that CKD was associated with high aPTT values, which are known to be associated with an increased risk of severe bleeding [12, 26]. It is possible that this is the result of impaired clearance of UFH secondary to CKD.

### Limitations

Several limitation to the present analysis deserve mentioning. First, in a significant proportion of patients the administered bolus dose heparin exceeded the currently recommended 70-100 IU/kg bolus dose [3, 4]. We used a fixed bolus dose UFH, corresponding to a wide variety of weight-adjusted doses, and this allowed us to investigate the effect of a broad variety of bolus doses on aPTT in patients with and without CKD. Moreover, the evidence in favour of the currently recommended bolus dose is scarce. In fact, in the present analysis, in as little as 16.8 % of patients in whom the recommended bolus dose was used the aPTT was in the recommended aPTT range, and in as many as 62 % of patients the aPTT was in excess of the recommended range. Second, the ACC/AHA currently recommend using ACT to monitor heparin treatment during PPCI [4]. However, it has been shown that aPTT is more closely related to heparin concentration in blood compared to ACT [27, 28]. Thus, aPTT is the appropriate measure to investigate if accumulation of heparin occurs in STEMI patients with CKD. Finally, as a result of the relatively small number of patients included in the analysis, it is possible that we could not detect significant differences in therapeutic, subtherapeutic high and markedly high aPTT ratios for patient with and without CKD, when different UFH bolus doses below 130 IU/kg were used.

## Conclusion

In STEMI patients with CKD undergoing PPCI, the risk of severe more persistent aPTT prolongation strongly increases with increasing heparin bolus, particularly beyond 130 IU/kg. Therefore, to reduce the risk of bleeding in these vulnerable patients, a UFH bolus dose below 130 IU/kg should be used. Adequately powered randomised controlled trials investigating the optimal UFH bolus dose in STEMI patients undergoing PPCI are needed.

## Electronic supplementary material

Supplementary material 1 (DOCX 1039 kb)
