# Supplementary material for: Influence of chronic kidney disease on anticoagulation levels and bleeding after primary percutaneous coronary intervention in patients treated with unfractionated heparin
Source: J Thromb Thrombolysis. 2015 Aug 4;41:441–51. doi: 10.1007/s11239-015-1255-x (PMC4799790; doi:10.1007/s11239-015-1255-x)
Supplement: Supplementary file 1 — Supplementary material 1 (DOCX 1039 kb) [file 11239_2015_1255_MOESM1_ESM.docx]

**Influence of chronic kidney disease on anticoagulation levels and bleeding after primary percutaneous coronary intervention in patients treated with unfractionated heparin**

*Wouter J. Kikkert^1^, MD, PhD, PhD, Peter M. van Brussel^1^, MD, Peter Damman^1^, MD, PhD, Bimmer E. Claessen^1^, MD, PhD, Jan P. van Straalen^2^, PhD, Marije M. Vis^1^, MD, PhD, Jan Baan Jr^1^,MD, PhD, Karel T. Koch^1^,MD, PhD, Ron J. Peters^1^, MD, PhD, Robbert J. de Winter^1^,MD, PhD, Jan J. Piek^1^, Jan G.P. Tijssen^1^,PhD, Jose P.S. Henriques^1^, MD, PhD*

*^1^* Department of Cardiology, Academic Medical Center – University of Amsterdam, Amsterdam, The Netherlands

^2^ Department of Clinical Chemistry, Academic Medical Center – University of Amsterdam, Amsterdam, The Netherlands

| Table 1. Baseline characteristics for patients in and excluded from the study | | | | |
| --- | --- | --- | --- | --- |
| Characteristic | | Included (n=1071) | Excluded (n=2401) | p-value |
| Male, n/N (%) | | 735/1071 (68.6) | 1737/2401 (72.3) | 0.025 |
| Age (years), mean (±SD) | | 61.8 (±13.2) | 61.7 (±13.0) | 0.91 |
| Bodymass index, median (IQR) | | 26.1 (24.1 - 29.0) | 26.0 (24.2 - 28.7) | 0.47 |
| History of, n/N (%) | |  |  |  |
|  | Diabetes | 143/1071 (13.4) | 302/2401 (12.6) | 0.53 |
|  | Hypertension | 388/1071 (36.2) | 804/2401 (33.5) | 0.12 |
|  | Hypercholesterolaemia | 238/1071 (22.2) | 548/2401 (22.8) | 0.70 |
|  | Current smoking | 491/1071 (45.8) | 990/2401 (41.2) | 0.011 |
|  | Previous MI | 118/1071 (11.0) | 299/2401 (12.5) | 0.23 |
|  | Previous PCI | 81/1071 (7.6) | 238/2401 (9.9) | 0.027 |
|  | Previous CABG | 17/1071 (1.6) | 69/2401 (2.9) | 0.024 |
|  | Family history CAD | 412/1071 (38.5) | 910/2401 (37.9) | 0.75 |
| Cardiogenic shock, n/N (%) | | 86/1066 (8.1) | 122/2351 (5.2) | 0.001 |
| IABP, n/N (%)^†^ | | 133/1069 (12.4) | 165/2396 (6.9) | < 0.001 |
| Glycoprotein IIb/IIIa Inhibitor, n/N (%) | | 143/1071 (13.4) | 828/2401 (34.5) | < 0.001 |
| Infarct related artery, n/N (%) | |  |  | 0.98 |
|  | RCA or LCx | 599/1048 (57.2) | 1235/2159 (57.2) |  |
|  | LAD or LM | 449/1048 (42.8) | 924/2159 (42.8) |  |
| Pre-procedural TIMI flow in IRA, n/N (%) | |  |  | 0.34 |
|  | 0/1 | 711/987 (72.0) | 1375/1955 (70.3) |  |
|  | 2/3 | 276/987(28.0) | 580/1955 (29.7) |  |
| Post-procedural TIMI flow in IRA, n/N (%) | |  |  | 0.45 |
|  | 0/1 | 29/1025 (2.8) | 70/2097 (3.3) |  |
|  | 2/3 | 996/1025 (97.2) | 2027/2097 (96.7) |  |
| Mulitvessel disease, n/N (%) | | 369/1042 (35.4) | 813/2166 (37.5) | 0.24 |
| Chronic total occlusion, n/N (%) | | 132/1042 (12.7) | 310/2166 (14.3) | 0.21 |

SD indicates standard deviation; IQR: interquartile range; MI: myocardial infarction; PCI: percutaneous coronary intervention; CABG: coronary artery bypass grafting; CAD: coronary artery disease; IABP: intra-aortic balloon pump; RCA: right coronary artery; LCx: left circumflex artery; LAD: left anterior descending artery; LM: left main artery; TIMI: thrombolysis in myocardial infarction; IRA: infarct related artery.


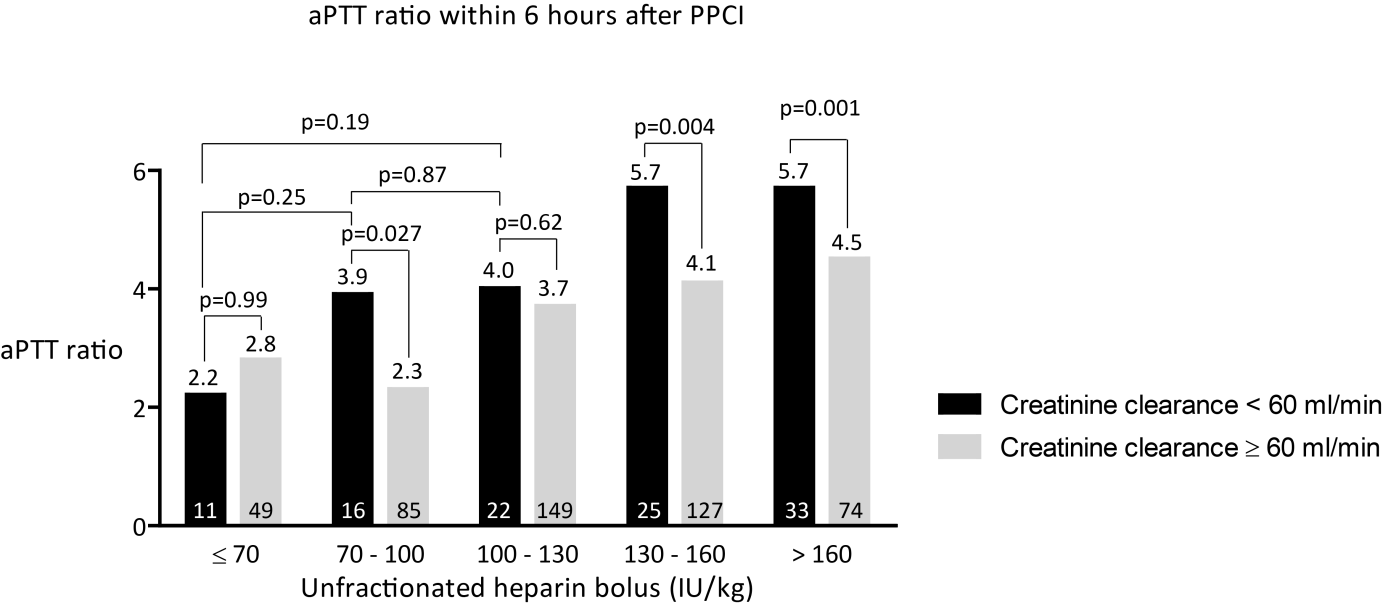


Figure 1. aPTT ratio determined between 6 and 12 hours after PPCI according to the administered heparin bolus for patients with and without CKD (creatinine clearance < 60 ml/min/1.73m^2^). Independent of the administered bolus dose heparin, aPTT ratio was higher in patients with CKD. Patients with CKD had higher aPTT ratios as compared to patients without CKD. Among patients with CKD, there was no statistically significant difference in aPTT ratio between patients treated with ≤ 70 IU/kg, 70 – 100 IU/kg, and 100 -130 IU/kg. The numbers at the bottom of the bars represent the number of patients in each group.


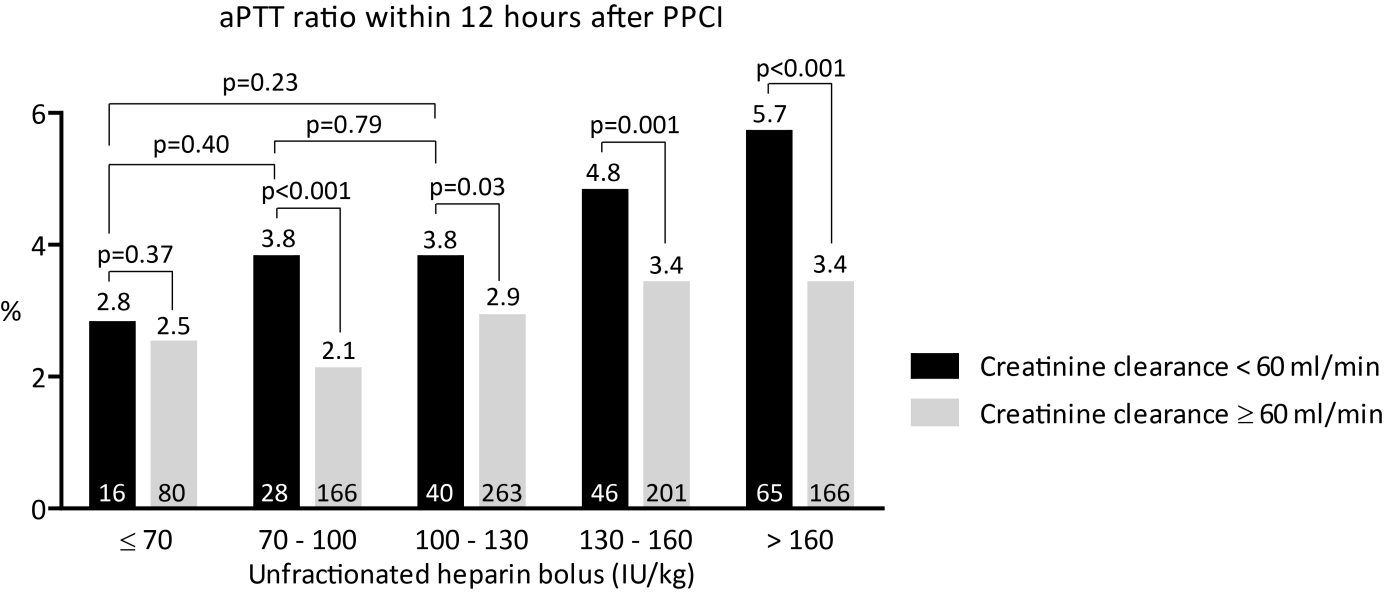


Figure 2. APTT ratio measured between the start of PPCI and 12 hours thereafter according to the administered heparin bolus for patients with and without CKD (creatinine clearance < 60 ml/min/1.73m^2^). Above 70 IU/kg, the aPTT ratio was higher in patients with CKD. The difference in aPTT ratio seemed to increase with increasing bolus. Among patients with CKD, there was no statistically significant difference in aPTT ratio between patients treated with ≤ 70 IU/kg, 70 – 100 IU/kg, and 100 -130 IU/kg (p≥0.23).


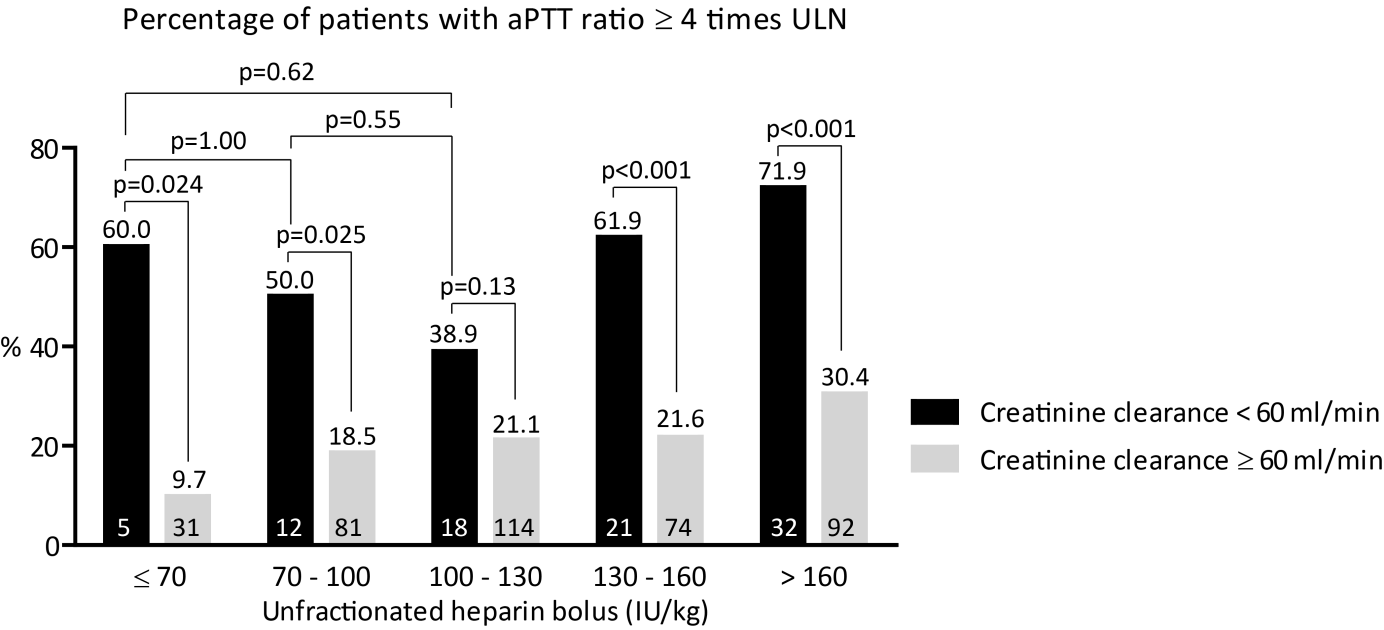


Figure 3. Percentage of patients with an aPTT ratio beyond 4 times control (measured between 6 and 12 hours after start of PPCI), as a function of unfractionated heparin bolus dose and creatinine clearance. For each heparin bolus dose, the percentage of patients with an aPTT ratio beyond 4 times control was higher if the creatinine clearance was below 60 ml/min. Among patients with CKD, there was no statistically significant difference in aPTT ratio between patients treated with ≤ 70 IU/kg, 70 – 100 IU/kg, and 100 -130 IU/kg (p≥0.62). The black and white numbers in the bars represent the number of patients in the respective patient group.


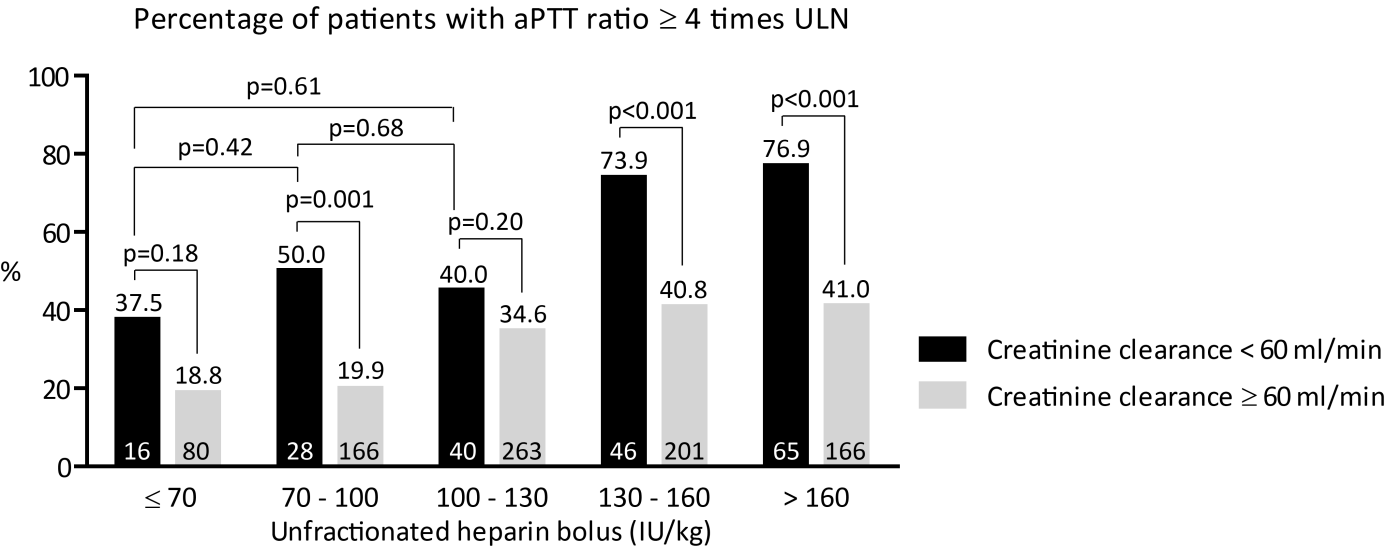


Figure 4. Supplementary figure 4. Percentage of patients with an aPTT ratio beyond 4 times control (measured the first 12 hours after start of PPCI), as a function of unfractionated heparin bolus dose and creatinine clearance. For each heparin bolus dose, the percentage of patients with an aPTT ratio beyond 4 times control was higher if the creatinine clearance was below 60 ml/min. The proportion of patients with markedly high aPTTs increased as the heparin bolus increased. With increasing heparin bolus there was a stronger increase in the proportion of patients with aPTT ratio ≥ 4 ULN in CKD as compared to no CKD. There was no statistically significant difference in severe aPTT prolongation between the patients with CKD treated with a ≤ 70 IU/kg, 70 – 100 IU/kg, or 100 – 130 IU/kg UFH bolus dose (p≥0.42). The black and white numbers in the bars represent the number of patients in the respective patient group.


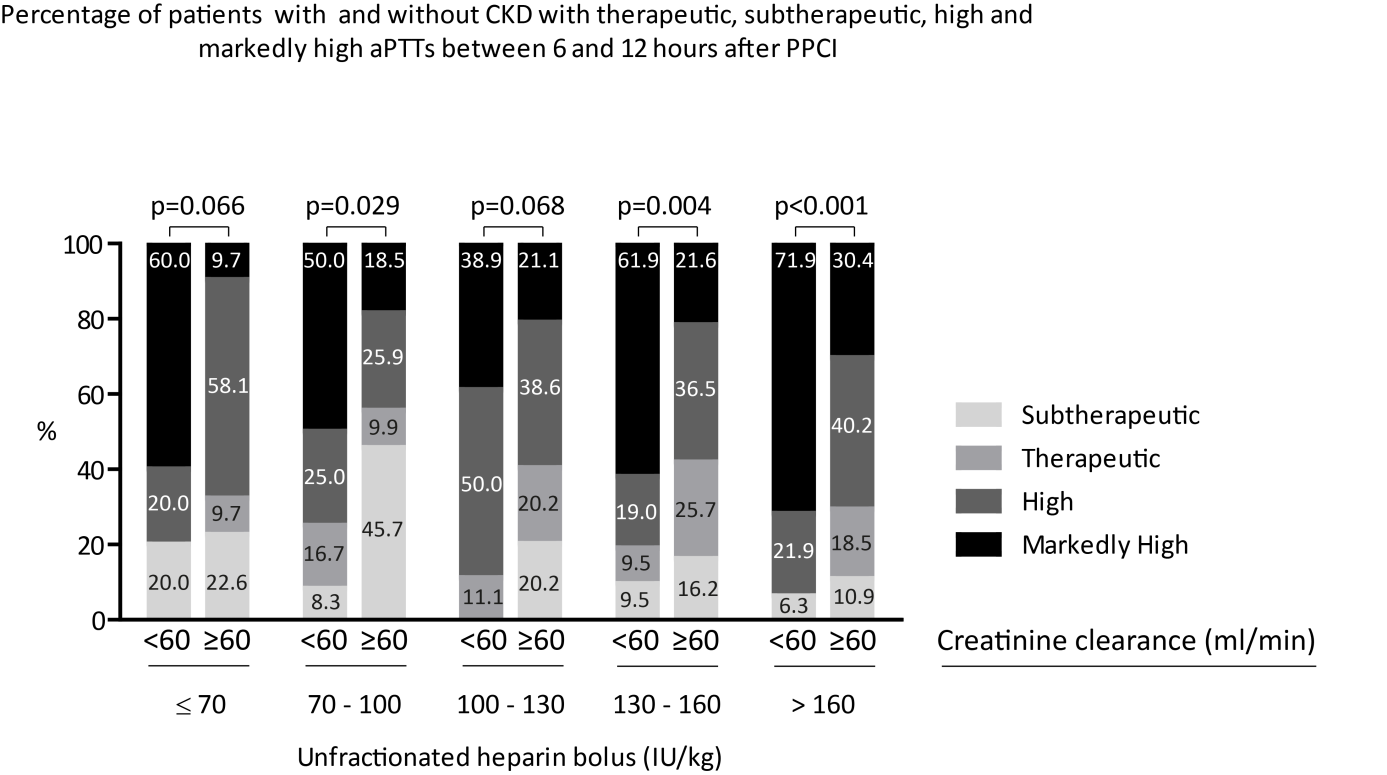


Figure 5. Percentage of patients with therapeutic, subtherapeutic, high and markedly aPTT ratios (measured between 6 and 12 hours after PPCI), as a function of unfractionated heparin bolus dose and creatinine clearance.


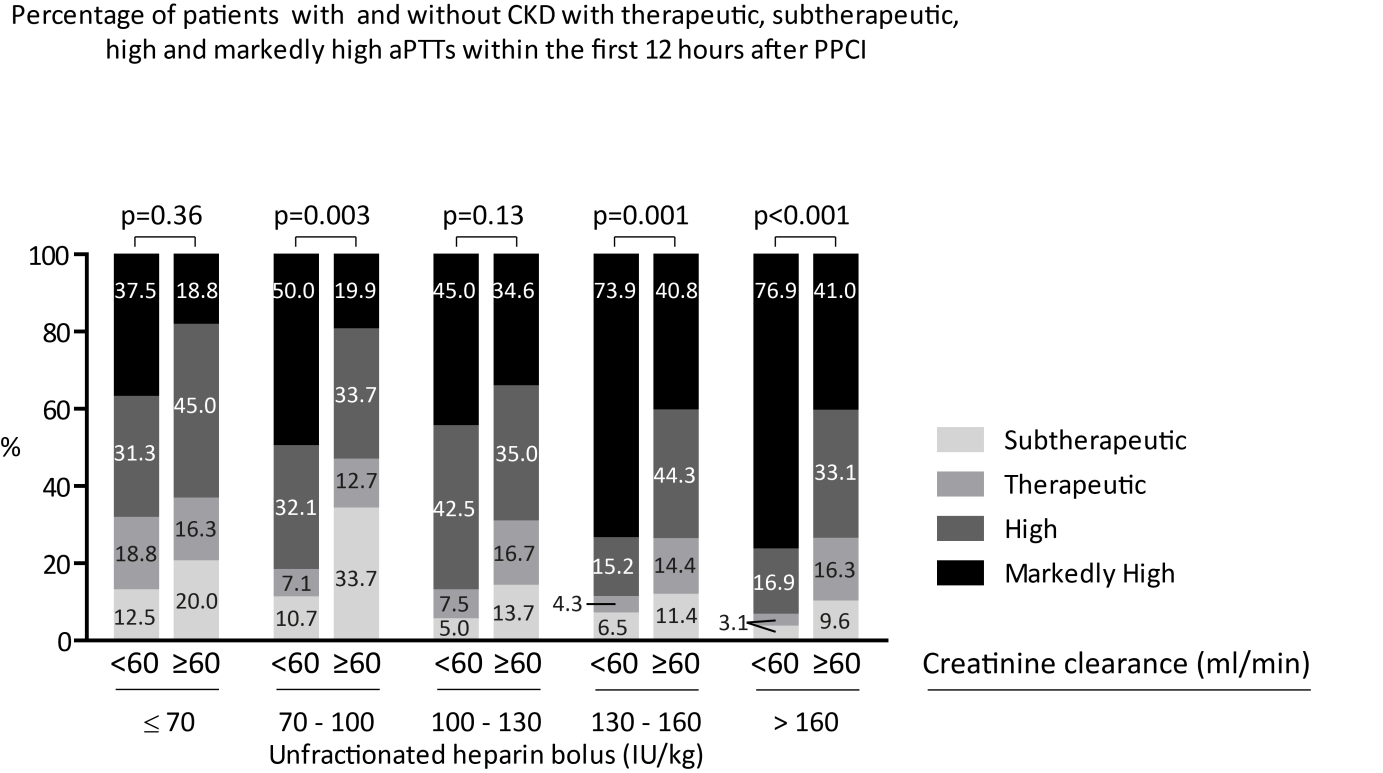


Figure 6. Percentage of patients with therapeutic, subtherapeutic, high and markedly aPTT ratios (measured between the start of PPCI and 12 hours thereafter), as a function of unfractionated heparin bolus dose and creatinine clearance. With increasing heparin bolus dose, the proportion of patients with high and markedly high aPTT ratios increased whereas the proportion of patients with subtherapeutic and therapeutic aPTT ratios decreased. This was more pronounced in CKD as compared to those without CKD.

**Table 2.** Relationship between creatinine clearance (per 10 ml/min decrement) and aPTT ≥ 4 ULN

|  | |  | Unadjusted | | | |  | Adjusted^a^ | | | |  |  | Adjusted^b^ | | | |  | |
| --- | --- | --- | --- | --- | --- | --- | --- | --- | --- | --- | --- | --- | --- | --- | --- | --- | --- | --- | --- |
| Hours after procedure | | n | OR | 95% CI | | p-value |  | OR | 95% CI | | p-value | p-value for interaction^c^ |  | OR | 95% CI | | p-value | | p-value for interaction^c^ |
| 6 hours^d^ | | 591 | 1.19 | 1.13 | 1.26 | < 0.001 |  | 1.14 | 1.05 | 1.24 | 0.001 | 0.21 |  | 1.11 | 1.05 | 1.18 | 0.001 | | 0.15 |
|  | < 130 IU/kg | 332 | 1.09 | 1.02 | 1.16 | 0.012 |  | 1.04 | 0.94 | 1.15 | 0.47 | 0.004 |  | 1.05 | 0.97 | 1.13 | 0.23 | | 0.002 |
|  | ≥ 130 IU/kg | 259 | 1.32 | 1.20 | 1.46 | < 0.001 |  | 1.38 | 1.19 | 1.60 | < 0.001 |  |  | 1.26 | 1.13 | 1.41 | < 0.001 | |  |
| 12 hours^e^ | | 815 | 1.31 | 1.23 | 1.39 | < 0.001 |  | 1.14 | 1.02 | 1.28 | 0.020 | 0.29 |  | 1.21 | 1.12 | 1.31 | < 0.001 | | 0.23 |
|  | < 130 IU/kg | 451 | 1.30 | 1.19 | 1.42 | < 0.001 |  | 1.20 | 1.02 | 1.40 | 0.025 | 0.57 |  | 1.24 | 1.11 | 1.39 | < 0.001 | | 0.55 |
|  | ≥ 130 IU/kg | 364 | 1.29 | 1.18 | 1.41 | < 0.001 |  | 1.08 | 0.92 | 1.28 | 0.35 |  |  | 1.17 | 1.04 | 1.31 | 0.007 | |  |
| First 12 hours^f^ | | 1071 | 1.25 | 1.19 | 1.31 | < 0.001 |  | 1.15 | 1.08 | 1.23 | < 0.001 | 0.58 |  | 1.15 | 1.09 | 1.20 | < 0.001 | | 0.48 |
|  | < 130 IU/kg | 593 | 1.19 | 1.11 | 1.26 | < 0.001 |  | 1.09 | 1.00 | 1.19 | 0.055 | 0.033 |  | 1.10 | 1.03 | 1.17 | 0.005 | | 0.019 |
|  | ≥ 130 IU/kg | 478 | 1.30 | 1.21 | 1.40 | < 0.001 |  | 1.27 | 1.14 | 1.41 | < 0.001 |  |  | 1.23 | 1.14 | 1.34 | < 0.001 | |  |

^a^ Calculated using multivariable logistic regression analyses adjusting for gender, bodymass, length, and heparin bolus dose.

^b^ Calculated using multivariable stepwise backward elimination logistic regression analyses including the following candidate covariables: gender, bodymass, length, heparin bolus dose, history of hypertension, diabetes, dyslipidemia, current smoking, stroke or TIA, peripheral artery disease, malignant disease, bleeding, recent surgery, previous MI, family history of CAD, anemia, leucocyte count, thrombocyte count, use of GP IIb/IIIa inhibitor, cardiogenic shock, and use of IABP.

^c^ p-value for the interaction term between heparin bolus dose (≥ 130 IU/kg versus < 130 IU/kg) and creatinine clearance (< 60 ml/min versus ≥ 60 ml/min)

^d^ Mean of APTTs determined between arterial sheath insertion and 6 hours hereafter.

^e^ Mean of APTTs determined between 6 to 12 hours after arterial sheath insertion.

^f^ Mean of APTTs determined between arterial sheath insertion and 12 hours hereafter.

APTT, activated partial thromboplastin time; CrCl, creatinin clearance

**Table 3.** Multivariable predictors of in hospital BARC type ≥ 3 bleeding

|  |  | OR | 95% CI | | p-value |
| --- | --- | --- | --- | --- | --- |
| Female gender | | 2.73 | 1.83 | 4.09 | < 0.001 |
| BMI, kg/m^2^ | |  |  |  | 0.11 |
|  | < 18.5 | 1.89 | 1.04 | 3.44 | 0.036 |
|  | 18.5 – 29.9 | 1.00 | - | - | - |
|  | ≥ 30 | 2.25 | 0.40 | 12.71 | 0.36 |
| GP IIB/IIIA inhibitor | | 1.90 | 1.14 | 3.18 | 0.014 |
| IABP |  | 3.37 | 2.11 | 5.39 | < 0.001 |
| Creatinine clearance | |  |  |  |  |
|  | < 60 ml/min/1.73 m2 | 2.77 | 1.81 | 4.26 | < 0.001 |
|  | ≥ 60 ml/min/1.73 m2 | 1.00 | - | - | - |
| Multivessel disease without concurrent CTO | | 1.60 | 1.02 | 2.52 | 0.040 |
| Multivessel disease with concurrent CTO | | 1.52 | 0.88 | 2.65 | 0.14 |

BARC indicates Bleeding Academic Research Consortium; OR: odds ratio; CI: confidence interval; GP: glycoprotein; IABP: intra-aortic balloon pump; CTO indicates chronic total occlusion.

**Table 4.** Multivariable predictor of in hospital major adverse cardiac events

|  | |  | OR | 95% CI | | p-value |
| --- | --- | --- | --- | --- | --- | --- |
| Family history of coronary artery disease | | | 0.40 | 0.23 | 0.69 | 0.001 |
| GP IIb/IIIa inhibitor | | | 1.69 | 0.95 | 2.98 | 0.073 |
| IABP | | | 2.56 | 1.47 | 4.45 | 0.001 |
| Cardiogenic shock | | | 1.78 | 0.93 | 3.41 | 0.081 |
| Creatinine clearance | | | |  |  |  |
|  | < 60 ml/min/1.73 m2 | | 2.52 | 1.55 | 4.08 | < 0.001 |
|  | ≥ 60 ml/min/1.73 m2 | | 1.00 | - | - | - |
| Anemia |  | | 2.04 | 1.23 | 3.38 | 0.006 |
| White blood cell count (x 10^9^/L) | | | |  |  |  |
|  | < 11 | | 1.00 | - | - | - |
|  | ≥ 11 | | 2.08 | 1.31 | 3.28 | 0.005 |
| Thrombocyte count (x 10^9^/L) | | |  |  |  | 0.033 |
|  | < 150 | | 3.41 | 1.44 | 8.07 | 0.005 |
|  | 150 - 400 | | 1.00 | - | - | - |
|  | > 400 | | 1.77 | 0.68 | 4.58 | 0.24 |
| Infarct related artery | | | |  |  |  |
|  | RCA/LCx | | 1.00 | - | - | - |
|  | LM/LAD | | 1.61 | 0.96 | 2.72 | 0.072 |
| Multivessel disease without concurrent CTO | | | 1.77 | 1.07 | 2.93 | 0.026 |
| Multivessel disease with concurrent CTO | | | 1.94 | 1.09 | 3.46 | 0.024 |

Predictors were identified with multivariable stepwise, backward elimination logistic regression analysis including the following candidate covariables:

RCA indicates right coronary artery; LCx: left circumflex artery; LM: left main artery; LAD: left anterior descending artery; other abbreviations as in table 2.
